# Development and Validation of a Low-Cost Arduino-Based Lee Disc System for Thermal Conductivity Analysis of Sustainable Roofing Materials

**DOI:** 10.3390/s25175447

**Published:** 2025-09-02

**Authors:** Waldemiro José Assis Gomes Negreiros, Jean da Silva Rodrigues, Maurício Maia Ribeiro, Douglas Santos Silva, Raí Felipe Pereira Junio, Marcos Cesar da Rocha Seruffo, Sergio Neves Monteiro, Alessandro de Castro Corrêa

**Affiliations:** 1 Electrical Engineering Program, Federal University of Pará—UFPA, Rua Augusto Corrêa, 01, Guamá CEP 66075-110, PA, Brazil; waldemiro.negreiros@ifpa.edu.br (W.J.A.G.N.); seruffo@ufpa.br (M.C.d.R.S.); 2 Materials Engineering Program, Federal Institute of Education, Science and Technology of Pará—IFPA, Avenida Almirante Barroso, 1155, Marco, Belém CEP 66093-020, PA, Brazil; jean.rodrigues@ifpa.edu.br (J.d.S.R.); alessandro.correa@ifpa.edu.br (A.d.C.C.); 3 Federal Institute of Education, Science and Technology of Pará—IFPA, Estrada do Icuí Guajará, Ananindeua CEP 67125-000, PA, Brazil; mauricio.maia@ifpa.edu.br; 4 Military Institute of Engineering—IME, Department of Materials Science, Praça General Tibúrcio, 80, Praia Vermelha, Urca, Rio de Janeiro CEP 22290-270, RJ, Brazil; raivsjfelipe@ime.eb.br (R.F.P.J.); sergio.neves@ime.eb.br (S.N.M.)

**Keywords:** thermal insulation, Lee Disc method, natural fiber composites, Arduino automation, low-cost instrumentation

## Abstract

The optimization of thermal performance in buildings is essential for sustainable urban development, yet the high cost and complexity of traditional thermal conductivity measurement methods limit broader research and educational applications. This study developed and validated a low-cost, replicable prototype that determines the thermal conductivity of roof tiles and composites using the Lee Disc method automated with Arduino-based acquisition. Standardized samples of ceramic, fiber–cement, galvanized steel, and steel coated with a castor oil-based polyurethane composite reinforced with miriti fiber (*Mauritia flexuosa*) were analyzed. The experimental setup incorporated integrated digital thermocouples and strict thermal insulation procedures to ensure measurement precision and reproducibility. Results showed that applying the biocompatible composite layer to metal tiles reduced thermal conductivity by up to 53%, reaching values as low as 0.2004 W·m^−1^·K^−1^—well below those of ceramic (0.4290 W·m^−1^·K^−1^) and fiber–cement (0.3095 W·m^−1^·K^−1^) tiles. The system demonstrated high accuracy (coefficient of variation < 5%) and operational stability across all replicates. These findings confirm the feasibility of open-source, low-cost instrumentation for advanced thermal characterization of building materials. The approach expands access to experimental research, promotes sustainable insulation technologies, and offers practical applications for both scientific studies and engineering education in resource-limited environments.

## 1. Introduction

The thermal efficiency of buildings has been consolidated as one of the main pillars for urban sustainability, driving technological advances in the selection and development of construction materials capable of minimizing energy losses and providing thermal comfort [1,2,3,4,5]. Roof tiles and coverings, in particular, play a critical role in regulating heat exchange between the external environment and the interior of buildings, significantly affecting energy consumption for air conditioning [6,7]. The appropriate selection of roofing materials therefore requires precise knowledge of their thermal properties, notably thermal conductivity (k), a parameter that determines the material’s ability to conduct heat [8,9,10,11].

In the scientific and technological context, the experimental determination of the thermal conductivity of construction materials remains a challenge, mainly due to the diversity of compositions, geometries, and operating conditions involved [12,13]. Conventional methods, such as the Guarded Hot Plate Method [14,15], the transient hot wire method [16], and thermal flux techniques [17,18], are considered reference standards due to their accuracy and reproducibility [19,20]. However, these techniques require sophisticated instrumentation, a controlled environment, rigorous sample preparation, and high financial investment, making their adoption restricted to large research centers and specialized laboratories [13,15,16]. Furthermore, their use in teaching and applied research environments encounters logistical and budgetary barriers [21,22,23].

The search for viable, low-cost, and easily replicable alternatives for measuring thermal conductivity in building materials has motivated the development of simplified and accessible experimental methods [21,22,23]. Among the classical approaches, the Lee Disc method stands out for its conceptual robustness, operational simplicity, and applicability in the characterization of insulating materials, such as tiles [23,24,25]. Traditionally, this method employs an analog apparatus, with manual measurements susceptible to human error and accuracy limitations [24,26]. The modernization and automation of the experiment, through integration with microcontrollers such as Arduino, represent an opportunity to democratize access to scientific instrumentation, increase accuracy, and enable open-source projects [24,25,26,27,28,29,30,31].

It is worth noting that the Lee disc method has greater applicability for materials with relatively low to moderate thermal conductivity, typically in the range of 0.1 to 5 W·m^−1^·K^−1^, including thermal insulators, polymers, composites, and ceramics. In this range, thermal equilibrium can be reproducibly achieved, and the assumptions of one-dimensional conduction remain valid. For materials with higher conductivity, above approximately 10 W·m^−1^·K^−1^, the technique tends to lose accuracy due to rapid heat dissipation and the increased influence of lateral losses and radiation [32,33]. This characteristic reinforces the suitability of the method for evaluating tiles and composite systems intended for thermal insulation in buildings.

The use of open platforms such as Arduino in experimental instrumentation has expanded in several areas. In education, studies have demonstrated its effectiveness in developing computational thinking in schools [27] and in teacher training through educational robotics [34]. In applied instrumentation, it has already been used in low-cost electrical conductivity measurement systems [28] and in work focused on prototyping smart sensors and actuators [30]. In civil engineering, recent initiatives incorporate digital solutions and machine learning for material characterization and monitoring [31]. Finally, in the context of sustainable composites, Negreiros et al. [35] demonstrated the potential of castor oil-based polyurethane matrices reinforced with miriti fibers as thermal insulation applied to metal tiles, which reinforces the relevance of the present study. Alazzawi et al. [36] presented a comparison between composites produced with palm fibers (DPF), hemp, and jute, evaluating their thermal and mechanical properties with a view to their application as insulating materials. Composite samples with different fiber mass fractions (10 to 30%) were prepared and molded under controlled conditions to ensure reproducibility. Characterization involved mechanical tests (compression, flexion, and impact) and thermal and chemical analyses (FTIR and DSC). The results showed that the thermal conductivity of the composites ranged from 0.0514 to 0.084 W/m^2^ K, being directly influenced by the incorporated fiber content, which reinforces their potential as sustainable construction materials.

Despite these advances, the literature highlights a significant gap related to the lack of low-cost, open, and easily replicable prototypes for measuring thermal conductivity in construction materials [23,24,25,26,27]. This lack limits the advancement of applied research, access to quality experimental training, and the prototyping of new materials in institutions with limited resources [26,27]. Developing innovative and accessible alternatives is, therefore, essential to promote the democratization of science and accelerate technological development in the construction sector [22,26,27,28].

Although the Lee Disc method has been reported for academic demonstrations and for basic characterization of low-conductivity materials, to the best of our knowledge, there is no prior work that integrates this technique into a fully automated, open-source, and low-cost prototype validated for construction materials. This study is original in two key aspects: (i) it democratizes access to reliable thermal conductivity measurements by using Arduino-based instrumentation, which drastically reduces costs compared to standard equipment; and (ii) it demonstrates, for the first time, the application of this approach to hybrid roofing systems incorporating natural fiber composites, particularly miriti fibers, relevant to the Amazonian context. By bridging scientific rigor with affordability and replicability, the present work fills a significant gap in experimental thermal characterization for both research and educational environments.

In this work, we developed a low-cost, easily replicable prototype for measuring the thermal conductivity of roofing materials, using the Lee disc method, automated through the Arduino platform. The system’s performance was experimentally validated by analyzing various representative samples of roofing materials used in the Amazon region, including ceramic tiles, fiber cement tiles, galvanized metal tiles, and metal tiles coated with a polyurethane/miriti (MPM) composite. All measurements were performed in a climate-controlled environment at a temperature of 24 ± 1 °C to minimize external variations and ensure the reproducibility of the results.

## 2. Materials and Methods

### 2.1. Materials

The thermal conductivity characterization of roofing materials was carried out through experimental tests using standardized samples, encompassing different constructive typologies and insulation materials, supported by high-precision instrumentation. The study focused on three categories of roofing tiles widely used in residential and commercial buildings in the Metropolitan Region of Belém, Brazil, selected based on their local market representativeness:

- Fiber Cement Tile (FCT): Samples purchased from the manufacturer Eternit S.A., standard corrugated model “Eternit Bigfort 6 mm”, in accordance with ABNT NBR 15575 [29]. The measured dimensions were 2.20 m long, 1.20 m wide, and 5 mm thick. The average density was approximately 1800 kg·m^−3^, and the nominal thermal conductivity provided by the manufacturer is 0.80 W·m^−1^·K^−1^.
- Ceramic Tile (CT): Commercial colonial-type samples provided by Cerâmica Ananindeua Ltd.a., identified as model “Colonial 40 × 23 cm”, also in accordance with ABNT NBR 15575 [29]. The measured dimensions were 0.401 m × 0.233 m × 0.010 m (length × width × thickness). The average density was 1500 kg·m^−3^, and the thermal conductivity was estimated between 0.70 and 1.05 W·m^−1^·K^−1^, according to the literature and manufacturer’s specifications.
- Galvanized Metal Tile (TG): Trapezoidal sheets of the TP-25 profile, supplied by Brasilit S.A., with dimensions of 1.80 m × 1.00 m and a thickness of 0.84 mm, confirmed with a digital caliper (accuracy 0.01 mm). Due to the high thermal conductivity of galvanized steel (≈52 W·m^−1^·K^−1^), this type of tile is usually used in conjunction with insulating materials, which was considered in subsequent analyses.
- Polyurethane/Miriti Fiber (MPM): A bi-component castor oil-based polyurethane system (IMPERVEG^®^ AGT 1315) reinforced with miriti palm fibers (*Mauritia flexuosa*), applied in the form of an insulating blanket. A fiber mass fraction of 10% was adopted, based on recent studies [33,35]. This sample was tested independently, in order to evaluate the intrinsic thermal behavior of the composite material.
- Metallic + MPM: Galvanized metal roofing sheets (TP-25 profile, supplied by Brasilit S.A.) coated on the inner surface with the polyurethane/miriti fiber composite described above. This hybrid condition was created to reduce the high thermal conductivity of bare metallic tiles (≈52 W·m^−1^·K^−1^) and represents a practical configuration for real roofing applications.

It is important to emphasize that the aluminum discs (D1–D4) described in Section 2.1.2 are not test samples, but integral parts of the Lee Disc apparatus. Their role is to provide a stable thermal pathway, to house the thermocouples, and to enable precise heat transfer measurements across the roofing material samples positioned between them.

The selected tiles and composites were tested under controlled laboratory conditions to ensure the reliability of the thermal data obtained. This detailed characterization was essential to support the assessment of the thermoenergetic performance of roofing systems under the climatic conditions of the Amazon region.

#### 2.1.1. Insulation and Composite Materials

To enhance the thermoenergetic performance of the metal roofing sheets, a bi-component vegetable-based polyurethane composite coating was applied. This adhesive system is derived from castor oil (*Ricinus communis*) and is commercially available under the IMPERVEG^®^ brand, specification AGT 1315. The composite was reinforced with natural fibers from the miriti palm (*Mauritia flexuosa*), sourced through collaboration with the Association of Miriti Producers of Abaetetuba, state of Pará, Brazil. Prior to application, the fibers underwent a controlled thermal treatment to eliminate residual moisture, aiming to prevent undesirable effects on the composite’s performance and to promote better interfacial adhesion between the fibers and the polymeric matrix [37]. A fiber mass fraction of 10% was adopted, in line with recent studies [35,36,37,38,39,40,41], and later confirmed by [42]. The use of thermal interface materials (TIMs) is highlighted in the literature as fundamental to reduce the thermal contact resistance between layers, contributing to greater measurement accuracy [38].

Additionally, a hybrid configuration, called Metallic + MPM, was prepared. This configuration consisted of a galvanized metal tile, TP-25 profile (Brasilit S.A., 0.84 mm thick), coated on its underside with the same castor oil-based vegetable polyurethane composite (IMPERVEG^®^ AGT 1315), reinforced with 10% by mass of miriti fibers (*Mauritia flexuosa*). The composite was manually applied by uniform spreading, followed by curing at room temperature for 48 h, ensuring adequate polymerization and adhesion to the metal surface. This configuration was included in the study because it represents a practical application of the composite as a thermal insulation layer on high-conductivity metal substrates, allowing us to evaluate the effective reduction in thermal conductivity in a multilayer system.

#### 2.1.2. Lee Prototype Components

**Figure 1:**
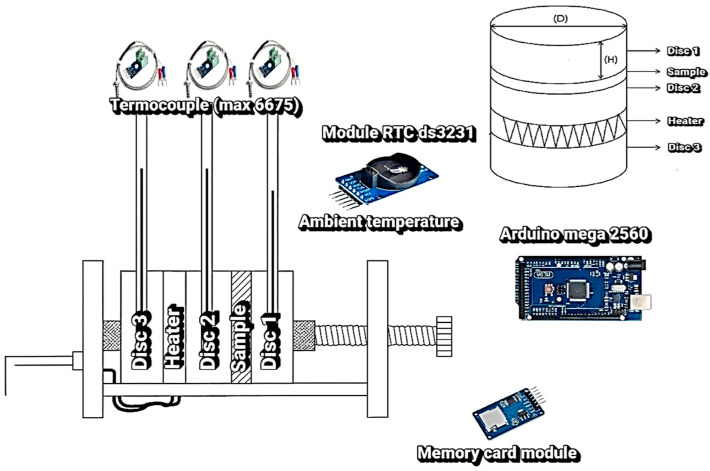
Experimental apparatus for the Lee Disc method. The system consists of three metal discs (Disc 1, Disc 2, and Disc 3), between which the sample of the material under study is inserted. A heater is positioned between Disc 2 and Disc 3 to promote heat flow. The temperature is monitored by K-type thermocouples coupled to MAX6675 modules. Temporal control is performed by the DS3231 RTC module, while data processing and storage are performed by the Arduino Mega 2560 board and the memory card module.

The experimental apparatus used for the thermal conductivity characterization of the samples (Figure 1) was based on the Lee’s Disc method [25], as refined in recent studies aimed at high-precision thermal measurements of low-conductivity materials [39]. This method was selected for its reliability, operational simplicity, and its ability to provide consistent results when applied to heterogeneous materials such as those used in roofing systems. The main components of the experimental setup were:

- Metal discs: Four aluminum discs were employed, each with a diameter of 10 cm and thickness of 1 cm. The discs were machined to a surface roughness below 0.8 µm to ensure optimal thermal contact with the samples. The mass of each disc was measured using a high-precision analytical balance (Shimadzu) with a resolution of 0.1 mg, ensuring accuracy in the thermal capacity calculations.
- Circular roofing samples: The samples were extracted using a diamond-tipped hole saw, maintaining a standardized diameter of 10 cm. The thickness corresponded to the original typology of each roofing material, and the edges were sanded carefully to correct surface imperfections and ensure adequate flatness, thereby minimizing thermal contact resistance between the layers.

To further ensure the precision of the setup, the discs were machined with uniform geometry (radius 25.5 mm, height 7.2 mm, and mass 39.3 g), providing thermal stability during testing. Three discs (D1, D2, and D3) were equipped with thermocouples for temperature monitoring, while the fourth disc (D4) was coupled to a ceramic heating resistor (nominal power 60 W, operating voltage 127 V), enabling stable and continuous thermal input.

D1 is the upper disc in direct contact with the sample (hot side, heat receiver for disc D2). D2 is the intermediate disc, instrumented with thermocouples, whose cooling rate is used in the conductivity calculation. D3 is the lower support disc, in contact with the opposite side of the sample, completing the conduction path, and D4 is the disc coupled to the ceramic resistor, responsible for supplying heat to the system.

The experimental setup was assembled and operated under controlled temperature and humidity conditions to ensure measurement reproducibility and minimize systematic errors.

#### 2.1.3. Instrumentation and Sensors

The instrumentation of the experimental apparatus was designed with an emphasis on automation and real-time data acquisition, using a platform based on the Arduino UNO microcontroller (Rev. 3, Arduino.cc), which has been widely validated in the literature for low-cost educational and scientific applications [28]. This choice enabled the integration of sensors, thermal control, and continuous data logging, ensuring reliability and reproducibility of the measurements. Temperature monitoring was performed using type K thermocouples, each installed at the central region of the aluminum discs (D1, D2, and D3) through threaded inserts filled with high thermal conductivity paste (Arctic MX-4). This configuration ensured proper thermal coupling between the sensors and the metallic substrates, minimizing spurious gradients across the disc surfaces. The thermocouples were connected to MAX6675 digital converter modules via SPI interface. Prior to testing, all sensors were calibrated against a reference digital thermometer (Testo 735-2, accuracy ±0.05 °C). After calibration, the system exhibited a combined uncertainty of ±0.1 °C, which is compatible with current best practices for thermal characterization experiments. The heating system consisted of a 10 W, 220 Ω ceramic resistor (manufacturer: Vishay, Malvern, PA, USA), powered by a regulated DC power supply (Minipa MPS-3003), which allowed stable thermal input throughout the experiments. The integration of sensors, thermal control, and automated data acquisition not only minimized human error in measurement readings, but also provided greater precision, repeatability, and traceability in thermal experiments. These advantages are consistent with recent developments in the use of embedded systems for low-cost thermal instrumentation [40,41], and subsequently validated in [42], reinforcing the feasibility of the adopted approach for scientific investigations in contexts with limited laboratory infrastructure.

#### 2.1.4. Sealing and Thermal Insulation Materials

In order to minimize thermal contact resistance between the metal discs and the samples, a thin layer of silicone-based thermal grease (Silicone Thermal Grease HY510) was applied, following recommendations from recent experimental protocols for measuring thermal conductivity in low-conductivity materials [39]. This thermal interface material enhances heat transfer between contact surfaces by mitigating the effects of surface micro-roughness and improving thermal coupling. Additionally, the experimental apparatus was externally insulated with a fiberglass blanket (ISOVER brand, 25 mm thick, thermal conductivity of 0.035 W·m^−1^·K^−1^) to prevent heat losses due to convection and radiation to the surrounding environment. This insulation strategy is aligned with best practices described in the scientific literature [41], ensuring greater thermal stability and measurement reliability throughout the testing process.

### 2.2. Methods

#### 2.2.1. Experimental Prototype Development

The experimental apparatus was developed based on the Lee’s Disc principle, widely employed for precise thermal conductivity measurements of solid insulating materials [39]. System control and data acquisition were managed using an Arduino Mega 2560 platform, integrated with an RTC DS3231 module for precise timing and a Micro SD Card module for continuous storage of experimental data. The electrical setup was carefully designed to ensure synchronized readings, data traceability, and experimental reproducibility, in accordance with recent recommendations for low-cost scientific prototyping [43,44].

**Figure 2:**
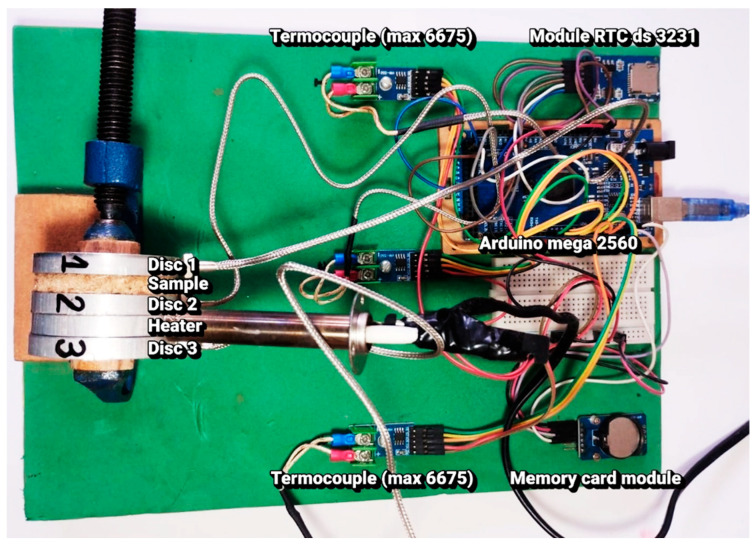
Experimental setup of the automated Lee’s Disc apparatus developed in this study. The system consists of aluminum discs (Disc 1, Disc 2, Disc 3) with the sample placed between Disc 1 and Disc 2, and a ceramic heater positioned between Disc 2 and Disc 3. Temperature acquisition was performed using type K thermocouples connected to MAX6675 converter modules. Data logging and control were managed by an Arduino Mega 2560 board, integrated with a real-time clock module (RTC DS3231) and a memory card module for storage.

All components were axially fastened using a 2″ C-clamp, ensuring uniform and consistent thermal contact between the discs and the samples during testing. The experimental assembly was placed in a climate-controlled environment, with the temperature maintained at 24 ± 1 °C, in order to reduce the influence of natural convection and ambient variations on the results, as recommended by recent thermal measurement protocols [45]. It is important to emphasize that all measurements reported in this study should be interpreted as thermal conductivities determined at 24 ± 1 °C, an ambient condition maintained throughout all tests. Figure 2 shows the developed prototype.

#### 2.2.2. Sample Preparation

**Figure 3:**
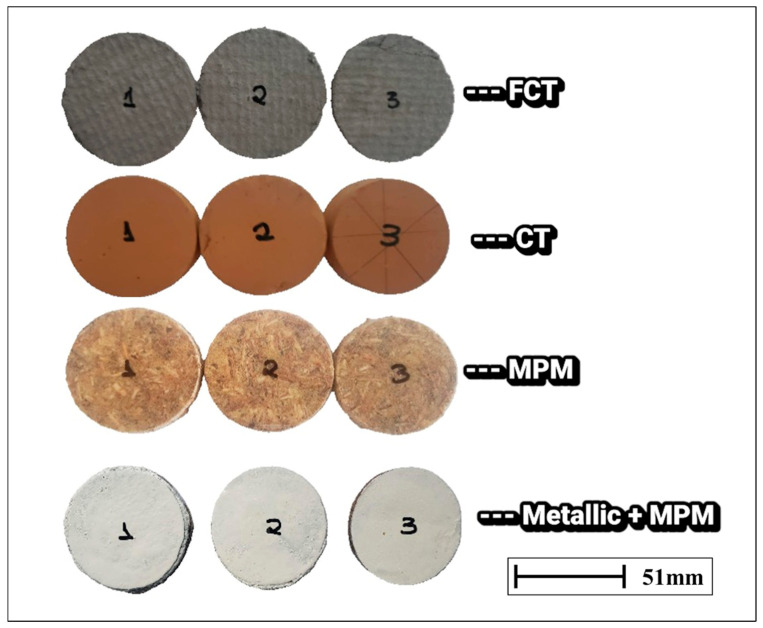
Circular samples of roofing materials prepared for thermal conductivity testing using the Lee’s Disc method. The groups represent: (i) Fiber cement tile (FCT); (ii) Ceramic tile (CT); (iii) Polyurethane/miriti fiber composite (MPM); and (iv) Metallic tile coated with polyurethane/miriti fiber composite (Metallic + MPM). Each set contains triplicate specimens (1–3) used to ensure reproducibility of the experimental results.

Circular samples were analyzed, obtained from different types of commercial roofing tiles, including ceramic, fiber cement, and metal tiles with thermal insulation blanket, as well as experimental prototypes composed of polymeric composites reinforced with natural fibers. The samples were carefully cut into discs with a diameter of 51.0 ± 0.1 mm, ensuring a precise fit between the aluminum discs of the experimental apparatus, thereby minimizing edge-related heat losses and ensuring the reliability of the thermal measurements [46]. The thickness of each sample was measured at three distinct points using a high-precision digital micrometer (Mitutoyo, 0.01 mm resolution), and the arithmetic mean of the values was used for analytical purposes. For each material condition, three independent samples (*n* = 3) were prepared and tested. Each sample was measured five consecutive times under identical conditions, generating multiple determinations of thermal conductivity. This procedure ensured statistical robustness and reproducibility of the results (Figure 3).

Table 1 presents the physical properties of the analyzed samples, while Table 2 contains the specifications of the metallic discs used in the experimental apparatus. The selection of materials was based on recommendations from the specialized literature, focusing on comparative studies of the thermal performance of conventional roofing materials and innovative composites, particularly those aimed at enhancing the thermoenergetic efficiency of buildings [35,47].

#### 2.2.3. Experimental Procedure

The experimental procedure strictly followed the protocols recommended by recent studies on thermal characterization using the Lee’s Disc method [16,41]. Initially, the sample was placed between discs D1 and D2, and the assembly was axially secured with a clamp, ensuring proper alignment and thermal contact among the components. Subsequently, disc D4, equipped with a heating element, was positioned above disc D3, configuring the system so that heat transfer occurred predominantly in a unidirectional manner, across the thickness of the sample.

The system was then energized, maintaining the heater under steady-state conditions until the temperature readings from the sensors in discs D1, D2, and D3 showed variations of less than ±0.5 °C over consecutive 3 min intervals (180 s), indicating that thermal equilibrium had been reached. Throughout the heating and cooling process, temperature data were automatically recorded every 3 min, following best practices for automated thermal data acquisition [47].

Once thermal steady state was achieved, the heater was turned off, and the cooling of the intermediate disc (D2) was monitored. The resulting cooling curve was used to determine the rate of heat loss by conduction, a fundamental step for the accurate calculation of the material’s thermal conductivity.

#### 2.2.4. Data Analysis

The physical principle underlying the Lee Disc method is based on one-dimensional steady-state heat conduction through a sample interposed between two high-capacity metal discs. During testing, the heater establishes a constant temperature gradient across the sample, and the conducted heat flux (*Q*) is described by Fourier’s Law (Equation (1)):

(1) $$Q=-kA\frac{\Delta T}{d}$$

where *k* is the thermal conductivity of the material, *A* is the cross-sectional area of the sample, *d* is its thickness, and Δ*T* is the temperature difference across its surfaces.

After the heater is switched off, the cooling of the intermediate disc is monitored. Its rate of temperature change (d*θ*/d*t*)*_c_*, combined with the heat capacity of the disc, allows the determination of the effective heat flux transmitted by the material. Therefore, the experimental procedure couples two complementary stages: (i) steady-state measurements to obtain Δ*T*, and (ii) transient cooling analysis to estimate *Q*. Together, these parameters constitute the basis for calculating thermal conductivity in the apparatus (Figure 1).

Following this principle, the determination of *k* in this study followed the analytical formalism of the Lee Disc method, widely validated for construction materials [16]. Initially, the steady-state heat transfer rate was calculated according to Fourier’s Law (Equation (2)):

(2) $$Q=\frac{k\cdot A\cdot (\theta_{2}-\theta_{1})}{d}$$

Rearranging, *k* can be isolated (Equation (3)):

(3) $$k=\frac{Q\cdot d}{A\cdot (\theta_{2}-\theta_{1})}$$

The heat transfer rate (*Q*) was estimated from the thermal capacity of disc D2 (*m*·*c*) and the cooling rate immediately after switching off the heater, as shown in Equation (4):

(4) $$Q=m\cdot c{\left(\frac{d\theta }{dt}\right)}_{c}$$

Thus, the final expression for the thermal conductivity is given by Equation (5):

(5) $$k=\frac{m\cdot c{\cdot (d\theta /dt)}_{c}\cdot d}{A\cdot (\theta_{2}-\theta_{1})}$$

It should be clarified that the thermal conductivity (*k*) values determined in this study are not associated with an isolated absolute temperature, such as *T*_2_, but rather with the temperature gradient established between the discs adjacent to the sample Δ*T* = (*T*_2_ − *T*_1_) in steady state. All measurements were performed in a climate-controlled environment at 24 ± 1 °C, so that the average effective temperature of the samples during testing remained close to the controlled room temperature. Thus, the *k* values reported in the results tables should be interpreted as thermal conductivities determined under steady-state room conditions, corresponding to average temperatures close to 24 °C.

This analytical procedure was applied to each experimental set, as discussed in [16], allowing rigorous comparison between the different materials tested. The statistical treatment considered the mean and standard deviation of the three replicates of each sample.

#### 2.2.5. Uncertainty Analysis

The accuracy of the thermal conductivity values obtained with the automated Lee’s Disc apparatus depends on several experimental parameters, such as sample thickness (*d*), temperature gradient between discs (Δ*T*), heat capacity of the aluminum disc (m·c), and the cooling rate (d*θ*/d*t*). To evaluate the reliability of the reported results, an uncertainty analysis was conducted considering the main sources of error:

- Temperature measurement: The K-type thermocouples coupled with MAX6675 converters presented a combined uncertainty of ±0.1 °C after calibration with a reference thermometer. For the observed gradients (30–70 K), this corresponds to a relative uncertainty below 0.3%.
- Sample thickness: Measured at three points with a digital micrometer (resolution 0.01 mm). For samples 5–12 mm thick, the relative uncertainty was estimated at ±0.2%.
- Disc mass and heat capacity: The aluminum discs were measured with ±0.1 mg precision, yielding negligible error (<0.1%). The adopted specific heat capacity (*c* = 9 × 10^2^ J·kg^−1^·K^−1^) may vary by ±2% depending on purity and processing, which was considered as a systematic contribution.
- Cooling rate determination: The slope of the initial cooling curve was obtained by linear regression of the first 60 s after heater switch-off. The standard deviation of the fit was typically below 2%.

By applying standard error propagation to Equation (5), the global uncertainty in the thermal conductivity (*k*) was estimated at ±5% (expanded, coverage factor *k* = 2). This result is consistent with the reproducibility observed in triplicate tests, in which the coefficient of variation (CV) remained below 5% for all samples. Therefore, the reported *k* values can be considered reliable within the stated uncertainty bounds, validating the feasibility of the low-cost automated prototype for comparative thermal characterization of roofing materials.

## 3. Results and Discussions

### 3.1. Prototype Validation and Experimental Performance

The experimental apparatus developed, based on the Lee’s Disc method and controlled via an Arduino platform, demonstrated adequate precision in thermal data acquisition, along with good reproducibility and stability throughout the tests performed. The integration of sensors with the automated system enabled continuous and reliable monitoring, ensuring efficient control of the experimental variables. The automation of the procedure significantly reduced human error in temperature and time readings, enhancing methodological rigor and allowing for the systematic repetition of experiments with high fidelity. This performance is consistent with best practices reported in the recent literature on low-cost scientific prototyping and educational applications for thermal measurements [16], highlighting the potential of the system for both applied research and technical training in thermal engineering.

**Figure 4:**
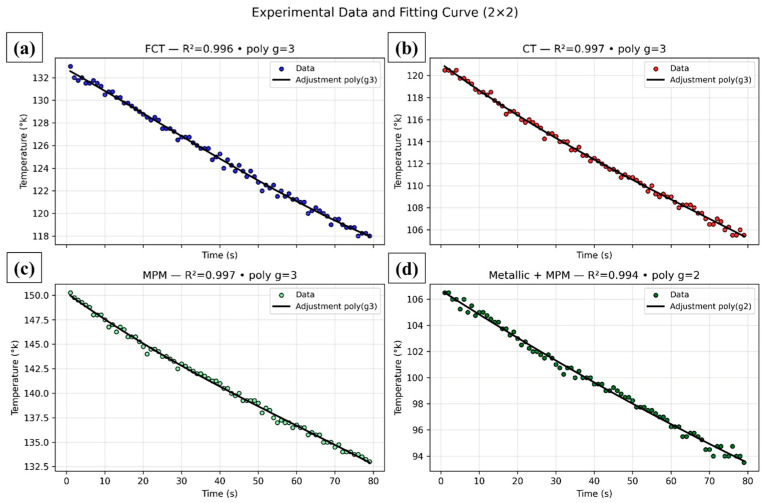
Experimental cooling curves obtained for samples of fiber cement tile (FCT) (**a**), ceramic tile (CT) (**b**), polyurethane/miriti fiber (MPM) (**c**) and metallic system coated with MPM (Metallic + MPM) (**d**), adjusted by polynomial regression in the initial region of thermal decay.

Figure 4 presents the representative cooling curves of the intermediate metal disc (D2) obtained for each of the analyzed samples: ceramic tile, fiber cement tile, Polyurethane/Miriti Fiber, and Galvanized metal tile coated with MPM.

Figure 4 presents the cooling curves obtained for the four evaluated conditions (FCT, CT, MPM, and Metallic + MPM), as well as the corresponding polynomial fits applied to the initial decay region. It can be observed that all fittings yielded very high determination coefficients (R^2^ > 0.994), evidencing the statistical robustness of the procedure. However, although the high correlation confirms the good empirical description of the data, it must be emphasized that the use of second- or third-degree polynomials is merely a mathematical approximation and does not directly reflect the physical mechanism of cooling. From a thermodynamic perspective, this phenomenon is classically represented by Newton’s Law of Cooling, which results in an exponential decay of temperature as a function of time. Thus, the application of exponential models would provide physically interpretable parameters, such as the thermal time constant (τ), making the analysis more meaningful.

Another relevant aspect concerns the visual interpretation of the curves. At first glance, the slopes appear similar among the different materials, but this impression is influenced by the distinct *Y*-axis scales adopted in each subplot, which may lead to a misleading perception. When analyzed numerically, the values of the initial cooling rate (d*θ*/d*t*) ranged from 0.1476 K·s^−1^ (MPM) to 0.2072 K·s^−1^ (Metallic + MPM), with intermediate values of 0.1899 K·s^−1^ (CT) and 0.1908 K·s^−1^ (FCT). Although these differences are subtle, they reveal thermal behaviors consistent with the nature of the materials: MPM exhibited the lowest cooling rate, confirming its performance as a better thermal insulator, while the Metallic + MPM system displayed the steepest initial slope due to the high thermal conductivity of the metallic substrate.

It is also important to highlight that, in Lee’s method, the thermal conductivity depends not only on the initial slope of the cooling curve but also on the sample thickness and the steady-state temperature difference (Δ*T*). Therefore, small variations in (d*θ*/d*t*), when combined with these additional parameters, become significant and lead to clearly distinct final conductivities among the materials. The values obtained were 0.429 W·m^−1^·K^−1^ for CT, 0.309 W·m^−1^·K^−1^ for FCT, 0.180 W·m^−1^·K^−1^ for MPM, and 0.200 W·m^−1^·K^−1^ for Metallic + MPM. These results confirm that the natural fiber-reinforced composites (CT and FCT) exhibit higher thermal conductivity compared to the mineral polymer matrix systems (MPM), thereby evidencing their superior capacity for thermal insulation.

In terms of thermal kinetics, the curves display an almost linear behavior within the 80 s interval analyzed, with a slight upward curvature, more pronounced in the MPM condition, indicating that the asymptotic equilibrium regime had not yet been reached. This characteristic reinforces the adequacy of exponential models for future analyses, as they would allow the identification of characteristic parameters of the heat transfer process. Moreover, complementary analyses such as residual diagnostics, information criteria (AIC and BIC), and confidence intervals for the fitting parameters would further enhance statistical robustness and avoid overfitting.

Therefore, the joint analysis of the experimental data and mathematical fittings demonstrates that, despite the satisfactory polynomial description, the physical interpretation is more consistent when associated with thermal parameters such as conductivity and time constant. In this context, it is noteworthy that the natural fiber-reinforced composites present clearly superior performance in terms of thermal insulation, whereas the incorporation of metallic substrates accelerates the cooling process due to their high conductivity but reduces insulation efficiency. These findings highlight the relevance of natural fibers as sustainable reinforcements in composite systems, both for improving thermal insulation and for their potential application in temperature-control devices and energy efficiency strategies.

### 3.2. Measuring the Thermal Conductivity of Roof Tiles

The thermal conductivity values presented in this section correspond to measurements performed in an air-conditioned environment at 24 ± 1 °C, the temperature at which the experimental apparatus was maintained during all tests. The results of the thermal conductivity (*k*) measurements are summarized in Table 3, which presents the mean values obtained for each sample, along with the corresponding standard deviations, calculated from three independent experimental replicates. For each material condition (CT, FCT, MPM, and Metallic + MPM), *n* = 3 independent samples were tested, each measured five times. The reported mean and standard deviation values therefore reflect the statistical treatment of these repeated measurements, ensuring reproducibility and reliability of the data.

Table 3 presents the experimentally obtained thermal conductivity (*k*) values for four different roofing and composite samples, determined using the Lee’s Disc method. The results reveal significant differences in the thermal behavior of the materials analyzed, with particular emphasis on the insulating performance of composites reinforced with natural Amazonian fibers.

The thermal conductivity values presented in Table 3 were obtained experimentally in this work using an automated Lee apparatus and were not adopted from the literature. All tests were conducted in a climate-controlled environment at 24 ± 1 °C, so the values of k should be interpreted as representative of this reference temperature condition.

The ceramic tile exhibited the highest thermal conductivity among all tested samples (k = (0.429 ± 0.012) W·m^−1^·K^−1^), a value consistent with the typical ranges reported in the international literature for dense ceramic roofing materials. Although widely used in residential roofing due to its low cost and high thermal inertia, this result indicates a greater capacity for heat conduction, which may lead to excessive indoor heat gain during the day—especially in hot and humid climates such as the Amazon region. Therefore, ceramic tiles alone may not provide adequate thermal comfort unless combined with additional strategies such as shading or forced ventilation.

The fiber cement tile exhibited a lower thermal conductivity than ceramic (k = (0.309 ± 0.009) W·m^−1^·K^−1^), reflecting its more diffusive nature and moderate thermal conduction capacity. These values are in agreement with literature reports for cementitious matrices reinforced with synthetic or natural fibers, placing fiber cement as an intermediate solution between thermal performance and economic feasibility. This explains its widespread use in low-income housing, with even greater efficiency when paired with insulating layers or ventilation systems.

Samples made from polyurethane (PU) composites reinforced with miriti fibers showed the lowest thermal conductivity values among all tested materials, confirming their effectiveness as thermal insulating materials. The MPM blanket, tested in isolation, reached (*k* = (0.180 ± 0.008) W·m^−1^·K^−1^), outperforming the other materials and aligning with values reported for polyurethane systems reinforced with natural fibers [35]. This performance highlights the synergistic effect between the polymeric matrix and natural fibers, which act as barriers to heat flow. In addition to its technical effectiveness, the use of miriti fibers (a renewable Amazonian resource) strengthens the environmental and economic viability of these composites in sustainable building applications.

The metal tile coated with the MPM blanket showed a slightly higher thermal conductivity than the blanket alone (*k* = (0.200 ± 0.007) W·m^−1^·K^−1^), as expected due to the high conductivity of the metallic substrate. Nevertheless, the composite layer was able to significantly reduce the effective thermal conductivity of the system, demonstrating its efficiency as a thermal barrier applied to the interior surface of the metal roofing. This result confirms that composite blankets, when used in multilayer configurations, represent a technically effective and economically attractive solution for improving the thermal performance of industrial, commercial, and residential buildings.

From a statistical perspective, the low standard deviations associated with all measurements confirm the high reproducibility of the experimental method and the thermal stability of the apparatus during testing. Furthermore, the differences between the mean values are sufficiently significant to demonstrate the superiority of natural fiber composites over conventional roofing materials in terms of resistance to heat conduction.

Thus, the results presented in Table 3 clearly demonstrate the technical and environmental feasibility of using polymeric composites reinforced with natural Amazonian fibers as efficient insulating materials. These composites exhibit competitive performance compared to conventional industrial solutions and align with the principles of sustainable construction. The partial or total replacement of high-conductivity materials with systems exhibiting lower k values can significantly contribute to the reduction in energy consumption for indoor climate control, particularly in regions with high thermal loads such as northern Brazil.

### 3.3. Critical Analysis and Comparison with Literature

**Figure 5:**
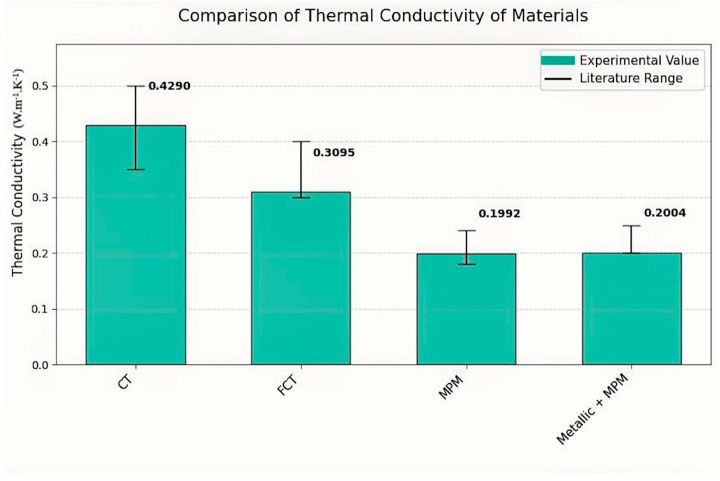
Comparison of experimentally determined thermal conductivity values for the analyzed samples: CT, FCT, MPM, and Metallic + MPM. Bars represent the mean values obtained with the Lee Disc method, while error bars indicate the typical ranges reported in the literature for equivalent materials.

Figure 5 presents a graphical comparison of the experimentally obtained thermal conductivity values for four different material configurations: ceramic tile (CT), fiber cement tile (FCT), Polyurethane/Miriti Fiber (MPM), and Galvanized metal tile coated with MPM (Metallic + MPM). The bars represent the mean thermal conductivity values in W·m^−1^·K^−1^, while the error bars indicate the typical range found in the literature for equivalent materials.

The figure provides a direct visual comparison between the experimental values and literature ranges, highlighting the relative thermal performance of each material. Table 4 complements this graphical analysis by detailing the numerical values, the reference temperature of the literature data, and the respective bibliographic sources, thereby ensuring precision and traceability.

It is worth highlighting again that the experimental values reported in this study correspond to measurements performed in a climate-controlled environment at 24 ± 1 °C, a condition maintained identically for all analyzed samples. The reference ranges extracted from the literature (Figure 5) correspond to values determined by different experimental approaches and technical compilations available for construction materials. In particular, Khomenko et al. [47] reported conductivities of englobed ceramics from conventional laboratory tests, while the Integrated Environmental Solutions database [48] presents tabulated data from standardized measurements and consolidated technical databases. Thus, although high-precision normative methods, such as the protected hot plate method (ASTM C177 [43]), are widely used in the literature for this type of material, it cannot be stated that they were specifically used in the references [47,48]; therefore, in this work, such ranges are treated only as comparative reference values. This methodological distinction should be considered when directly comparing the results, although the orders of magnitude and ranges of variation show good agreement.

The results reveal clear trends: polymeric composites reinforced with natural fibers lead to a significant reduction in thermal conductivity, offering superior thermal performance compared to traditional materials. The MPM blanket, for example, achieved the lowest conductivity value among all tested samples (0.180 ± 0.008) W·m^−1^·K^−1^, representing a reduction of over 58% compared to conventional ceramic tile. This performance is comparable to that of polyurethane composites reinforced with other plant fibers, such as curaua or coir [35].

The ceramic tile exhibited the highest thermal conductivity value (0.429 ± 0.012) W·m^−1^·K^−1^, indicating a high capacity for heat conduction. While widely used in residential roofing due to its availability and low cost, this result suggests that, in hot and humid regions such as the Amazon, ceramic tiles used alone may be insufficient to ensure adequate thermal comfort—particularly in the absence of complementary solutions such as shading or ventilation.

The fiber cement tile presented an intermediate thermal conductivity value (0.309 ± 0.009) W·m^−1^·K^−1^, also consistent with the literature for cementitious matrices reinforced with synthetic or natural fibers. Its slightly better thermal performance compared to ceramic supports its use in social housing, although additional solutions are needed when aiming for higher energy efficiency in buildings.

The samples composed of polyurethane composites with miriti fibers exhibited the best thermal insulation performance. The standalone MPM blanket demonstrated high potential as an insulating material, due to the low intrinsic conductivity of the polymer matrix and the role of the natural fibers, which introduce heterogeneities that hinder heat conduction. The metal tile with composite blanket showed a slightly higher conductivity value (0.200 ± 0.007) W·m^−1^·K^−1^, yet still significantly lower than typical values for uninsulated metal roofing. This confirms that the application of a composite blanket as an internal lining can effectively reduce the thermal conductivity of metal systems.

Error bars from the literature ranges validate the experimental data, as all mean values fall within expected intervals, confirming the reliability of the Lee’s Disc method and the measurement system. Furthermore, the low standard deviations observed between samples indicate high reproducibility of the tests and adequate control of experimental variables.

Overall, the data presented in Figure 5 reinforce the technical and environmental viability of natural fiber composites as effective thermal alternatives to conventional roofing materials. The use of the MPM blanket, both as a standalone layer and as an inner lining for metal tiles, proves highly promising for applications in buildings located in regions with high thermal loads. Additionally, the results suggest potential for use in retrofitting strategies, especially for existing metal roofs, enabling improved thermal performance through low-cost interventions with reduced environmental impact. The behavior observed for the composites is consistent with recently developed systems aimed at energy efficiency in buildings, as reported by other authors. Table 4 shows the comparison of the thermal conductivity values of the experimental samples and typical ranges from the literature.

Based on the comparative thermal conductivity data presented in the table, it is possible to clearly and thoroughly assess the relative thermal efficiency of the tested materials, as well as validate the experimental results against literature references. The findings demonstrate that all analyzed samples exhibited thermal conductivity values consistent with previously reported ranges, underscoring the reliability of the adopted methodology and the quality of the conducted tests.

It is important to emphasize that the values presented in the “Experimental value” column were determined exclusively in the present work, using the automated prototype based on the Lee Disk method, under controlled conditions of 24 ± 1 °C. The “Literature range” column corresponds to typical thermal conductivity ranges reported by other authors in materials of similar composition, serving only as a comparative and validation reference.

In the case of ceramic roof tiles (CT), Khomenko et al. [47] reported conductivities between 0.35 and 0.50 W·m^−1^·K^−1^ in dense englobed ceramics, which are representative of traditional tiles. For fiber cement roof tiles (FCT), the range of 0.30–0.40 W·m^−1^·K^−1^ was obtained from the technical database of Integrated Environmental Solutions [48], which compiles normative and experimental values for asbestos-free fiber cement boards. In the case of polyurethane composites reinforced with vegetable fibers, Negreiros et al. [35] reported conductivities of 0.18–0.24 W·m^−1^·K^−1^ for the PU/miriti (MPM) composite tested in isolation and of 0.20–0.25 W·m^−1^·K^−1^ when applied as a coating on metallic substrates. These values were obtained in independent experiments, based on normative methods such as hot-plate and heat flux.

Therefore, the ranges presented in Table 4 were not used directly in the calculations of this work, but merely as reference ranges to validate the experimental values measured. This procedure reinforces the consistency of the results and their adherence to the state of the art in thermal characterization of building materials.

These results highlight the effectiveness of the MPM composite coating in mitigating heat conduction in highly conductive substrates such as metal, representing up to a 53% reduction in heat transfer compared to conventional ceramic tiles. This performance reinforces the potential of combining biobased polyurethane with Amazonian natural fibers as a viable and efficient solution for roofing systems in tropical climates, contributing significantly to thermal comfort and energy efficiency. Additionally, the close agreement between experimental values and literature ranges validates the adopted methodology and confirms the robustness of the findings achieved in this study.

### 3.4. Limitations and Experimental Sensitivity Analysis

Despite the good performance demonstrated by the experimental apparatus and the consistency of the results obtained, it is important to acknowledge the inherent limitations of the adopted method and to critically assess the sensitivity of the experimental variables involved. This approach is essential to ensure accurate interpretation of the data, demonstrate methodological consistency, and outline pathways for future methodological improvements.

In this study, the classical formalism of the Lee method was adopted, which is based on a one-dimensional steady-state heat transfer model. This hypothesis is suitable for homogeneous, low-conductivity materials, but presents limitations when applied to multilayer systems with significant contrasts in thermal properties, as in the case of the metal tile coated with MPM membrane. In these configurations, the high conductivity of the metal substrate can favor lateral heat diffusion, partially deviating from the ideal conditions of purely axial conduction. Although the experimental apparatus was designed to reduce these effects through geometric standardization of the samples, application of thermal grease to minimize contact resistance, and peripheral insulation with a fiberglass membrane, it is not possible to completely eliminate the contribution of lateral flows in heterogeneous materials. This factor may partially explain the nonlinearity observed in the cooling curve of the metal + MPM system. It should be noted, however, that the conductivity calculation was based on the initial cooling rate of the intermediate disc, a region in which deviations due to lateral diffusion mechanisms and parasitic losses are minimized, ensuring the representativeness of the values obtained.

Another important limitation concerns the insufficient control over external heat losses. Although the apparatus was insulated with a fiberglass blanket and shielded to minimize convection and radiation, the real efficiency of this insulation was not quantified. The lack of precise measurement of the external heat leakage may introduce deviations in the determination of the thermal conductivity coefficients, especially for materials with low intrinsic conductivity. Recent studies also emphasize that uncontrolled boundary conditions and partial insulation can significantly influence the accuracy of thermal transport measurements in composite and porous systems [49,50,51].

Another critical factor is the need to reach and maintain thermal steady state during the tests. The accurate identification of this equilibrium point depends on the stability of the measured temperatures, which may be affected by electronic noise, environmental fluctuations, or power supply instability. The use of an automated data acquisition system based on Arduino and digital sensors helped minimize human error, but did not eliminate external influences entirely.

Sample preparation also plays a key role in measurement reliability. Standardized circular discs (51.0 ± 0.1) mm in diameter were used, with edge sanding and the application of high-conductivity thermal grease (HY510) to reduce thermal contact resistance. However, imperfections in surface flatness, non-uniform clamp pressure, and micro-gaps at the interfaces may have caused localized thermal resistance, affecting the desired heat transfer pathway. While such sources of error were mitigated, they are difficult to eliminate entirely.

In terms of instrumentation, the system employed Arduino Mega 2560, type K thermocouples, and MAX6675 digital converter modules—components widely validated for low-cost scientific prototyping. Despite their proven effectiveness, the system has limited resolution (10-bit ADC) and is susceptible to electromagnetic noise and signal fluctuations. Sensor calibration with a reference digital thermometer (Testo 735-2, ±0.05 °C accuracy) and averaging of multiple readings helped reduce uncertainty, but residual noise remained a limiting factor.

The experimental sensitivity analysis revealed that thermal conductivity (*k*) is particularly sensitive to two critical parameters: sample thickness (L) and temperature variation in the steady-state regime (Δ*T*). Small measurement errors in these variables directly impact the final k value. Therefore, a high-precision digital micrometer (0.01 mm resolution) was used, and the experiments were conducted under strict thermal stability criteria, with temperature variation maintained below ±0.5 °C in three-minute intervals. Additionally, the thermal capacity of the metal discs, a function of mass and specific heat, was found to directly influence the cooling rate of the intermediate disc (D2), which is a key parameter in the calculation of heat loss and, subsequently, thermal conductivity.

Another relevant point concerns the preparation and characterization of the composite samples. Thickness uniformity was not fully verified; only the average value obtained from three spot measurements with a digital micrometer was used. Although this procedure is sufficient for applying the Lee method to low-cost prototypes, the lack of the associated standard deviation and a more detailed analysis of surface morphology constitutes a limitation of this study. Local variations in thickness, roughness, or flatness imperfections can introduce additional thermal contact resistances, affecting the ideal linearity of the conduction process. Future investigations should include additional characterization methods, such as scanning electron microscopy (SEM), profilometry, and more robust statistical analyses, in order to more accurately quantify these effects.

Additionally, it was observed that the cooling curve of the sample composed of a polymeric mat reinforced with miriti fibers (MPM) exhibited nonlinear behavior over time. This effect can be attributed to the intrinsic structural heterogeneity of the composite, in which the irregular distribution of fibers in the polymer matrix generates differentiated conduction paths and multiple time constants in the dissipation process. Small variations in thermal contact resistance, even after the use of conductive grease and standardized axial tightening, can also contribute to this behavior. Nevertheless, the thermal conductivity determination was performed based on the initial cooling rate, a region in which the assumptions of the Lee method remain closest to ideal conditions and the parasitic effects of convection and radiation are minimized, ensuring the reliability of the values obtained.

Even with these limitations, the results showed high reproducibility, with a coefficient of variation (CV) below 5% for all tested samples, reinforcing the robustness of the adopted method. The mean thermal conductivity values remained within the typical ranges reported in the literature for equivalent materials, further validating the reliability of the experimental protocol.

It is also important to highlight that the Lee Disc method, although robust and cost-effective, represents a limit case when applied to multilayer systems such as the metallic tile coated with the MPM composite. In these configurations, the sharp contrast in thermal properties between the metal substrate (high conductivity) and the composite layer (low conductivity) can introduce lateral heat diffusion and multiple dissipation time constants, partially deviating from the one-dimensional conduction assumption of the method. While the present study minimized such effects by using conductive grease, controlled clamping, and peripheral insulation, future research should integrate numerical simulations (2D/3D heat transfer models) to complement experimental results and better capture the complexity of multilayer thermal systems.

When contextualized with the data available in the literature, the experimental results obtained in this study show strong agreement with the ranges previously reported for equivalent materials. The measured thermal conductivity for the ceramic tiles (0.429 W·m^−1^·K^−1^) is within the range of 0.35–0.50 W·m^−1^·K^−1^ described by Khomenko et al. [47] for high-density englobed ceramics. Similarly, the fiber cement tile presented 0.3095 W·m^−1^·K^−1^, a value that falls directly within the range of 0.30–0.40 W·m^−1^·K^−1^ compiled by Integrated Environmental Solutions [48].

In the case of polyurethane-based composites reinforced with miriti fibers, the measured values of 0.1992 W·m^−1^·K^−1^ (MPM) and 0.2004 W·m^−1^·K^−1^ (Metallic + MPM) are in full agreement with the ranges of 0.18–0.24 W·m^−1^·K^−1^ and 0.20–0.25 W·m^−1^·K^−1^, respectively, reported by Negreiros et al. [35] in normative tests. This agreement is particularly relevant because it validates the reliability of the prototype developed in this work when compared with traditional high-precision techniques, demonstrating its effectiveness as a low-cost alternative for measuring thermal conductivity.

Overall, the close correspondence between the values obtained here and the literature reference ranges reinforces the methodological robustness of the proposed system and highlights the potential of natural composites as sustainable thermal insulators. In addition to demonstrating the accuracy of the experimental apparatus, the results highlight the technical and environmental feasibility of using Amazonian fibers in construction systems, contributing to energy-efficient solutions in tropical climates.

However, the polyurethane/miriti fiber composite (0.180–0.200 W·m^−1^·K^−1^) presents a clear advantage, situating itself near the lower bound of insulating materials employed in tropical building envelopes [35]. This substantial reduction in *k* is particularly significant for the Amazonian context, where high solar incidence and elevated ambient temperatures demand effective, low-cost solutions for reducing heat gain indoors. Therefore, the proposed system not only validates the Lee Disc apparatus as a scientific tool but also provides practical guidance for sustainable construction strategies adapted to tropical climates.

## 4. Conclusions

This study has demonstrated the technical feasibility and functional effectiveness of a low-cost, replicable, and automated prototype, based on the Arduino platform, for the accurate measurement of thermal conductivity in roofing materials and composites applied to civil construction. The adopted approach integrated digital sensors, automated data acquisition, and Arduino-based processing, resulting in a robust and stable experimental system that offers high reproducibility of the measurements.

The originality of this work lies not only in the design of a low-cost prototype but also in its validation against conventional roofing materials and innovative natural fiber composites. Unlike previous studies, the present research provides a replicable, open-source methodology that combines experimental accuracy with affordability, enabling laboratories with limited resources to perform advanced thermal characterization. Moreover, the application of miriti fiber composites in roofing systems highlights a unique contribution to sustainable construction materials research, particularly tailored to tropical regions. This dual innovation, instrumental and material, reinforces the novelty and relevance of the proposed approach.

Among the key findings, particular emphasis is placed on the superior performance of the metal roofing panel coated with a biocompatible composite made of bio-based polyurethane reinforced with miriti fibers (*Mauritia flexuosa*), which reduced the thermal conductivity from a typical value of 52 W·m^−1^·K^−1^ (for the bare metal) to 0.2004 W·m^−1^·K^−1^. This represents a reduction of up to 53% in heat transfer, surpassing the thermal performance of commercial fiber cement 0.3095 W·m^−1^·K^−1^ and ceramic tiles 0.4290 W·m^−1^·K^−1^, and highlighting the potential of such composites to enhance thermal comfort in buildings located in tropical climates, such as the Amazon region.

The main contribution is the design and validation of an accessible, replicable methodology suitable for both resource-constrained research and educational laboratories. By democratizing access to thermal characterization techniques through low-cost scientific instrumentation, this study aligns with contemporary trends in open science, hands-on learning, and sustainable development.

Moreover, the results reinforce the viability of natural and hybrid composites as effective and environmentally responsible alternatives for thermal insulation, especially in buildings aiming for energy efficiency and reduced carbon footprint. The versatility of the developed apparatus allows for its customization to study different classes of materials, including polymers, biomaterials, and technologically relevant composites.

Nonetheless, some limitations must be acknowledged. Despite the good accuracy demonstrated, the system may still be sensitive to uncontrolled environmental variations, thermal contact resistance, and sensor resolution limitations. These factors point to the need for future improvements, such as enhanced thermal insulation, the use of higher-resolution sensors, and the implementation of more rigorous calibration protocols. Additionally, further studies should investigate the durability and performance of the composites under prolonged thermal cycling and exposure to real-world environmental conditions, to validate their practical applicability.

In summary, this research represents a meaningful contribution at the intersection of materials science, sustainability, and innovation in thermal instrumentation, opening new pathways for material characterization in both academic and applied research contexts. The continued enhancement of this approach can significantly contribute to the training of better-prepared professionals, as well as to the dissemination of more efficient, sustainable, and cost-effective construction technologies, especially in regions facing high climatic and socioeconomic vulnerability.

## Figures and Tables

**Table 1:** Experimental parameters of FCT: fiber cement tile; CT: ceramic tile; MPM: polyurethane/miriti fiber; Metallic + MPM: TP-25 galvanized metal tile coated with MPM blanket.

Parameters	FCT	CT	MPM	Metallic + MPM
Thickness (mm)	4.8	11.6	7.2	6.4
Cooling rate (K·s^−1^)	0.1908	0.1899	0.1476	0.2072
Stationary temperature difference (T2-T1) (K)	31.25	54.25	59.50	70

**Table 2:** Data from the metal disc used in the experiments.

Parameter	Value
Mass (kg)	0.0393
Height (mm)	7.2
Heat capacity of aluminum (J·kg^−1^·K^−1^)	9 × 10^2^
Radius (mm)	25.5

**Table 3:** Average thermal conductivity values (*k*) of the samples evaluated by the Lee disc method, determined in an air-conditioned environment at 24 ± 1 °C.

Sample	k [W·m^−1^·K^−1^]	Standard Deviation
CT	0.429	0.012
FCT	0.309	0.009
MPM	0.180	0.008
Metallic + MPM	0.200	0.007

**Table 4:** Comparison between experimental thermal conductivity values obtained in this study (24 ± 1 °C) and typical ranges reported in the literature for equivalent materials under conditions close to room temperature.

Material	Experimental Value (W·m^−1^·K^−1^)	Literature Range (W·m^−1^·K^−1^)	References
CT	0.429	0.35–0.50	[47]
FCT	0.3095	0.30–0.40	[48]
MPM	0.1992	0.18–0.24	[35]
Metallic + MPM	0.2004	0.20–0.25	

## Data Availability

The original contributions presented in the study are included in the article; further inquiries can be directed to the corresponding author.
